# Synergistic modulation of proinflammatory mediators and cytokines in lipopolysaccharide-activated RAW 264.7 macrophages: The therapeutic potential of *Elephantopus scaber* and *Sauropus androgynus* ethanol extract

**DOI:** 10.14202/vetworld.2024.728-734

**Published:** 2024-03-25

**Authors:** Muhammad Sasmito Djati, Yuyun Ika Christina, Dinia Rizqi Dwijayanti, Sri Rahayu

**Affiliations:** 1 Department of Biology, Faculty of Mathematics and Natural Sciences, Brawijaya University, Malang 65145, East Java, Indonesia; 2 Dewan Jamu Indonesia East Java Region, Malang 65145, East Java, Indonesia

**Keywords:** *Elephantopus scaber*, inflammation, macrophage, nitric oxide, proinflammatory cytokines, *Sauropus androgynous*

## Abstract

**Background and Aim:**

*Elephantopus scaber* (ES) and *Sauropus androgynus* (SA) have broad biological effects and have long been used in traditional medicine. However, the anti-inflammatory properties of the combination of ES and SA have not yet been fully explored. This study aimed to investigate the anti-inflammatory activities of the combination of ES and SA ethanol extract on lipopolysaccharide (LPS)-activated RAW 264.7 macrophage cell lines by inhibiting proinflammatory mediators and cytokines.

**Materials and Methods:**

Nitric oxide (NO) production in RAW 264.7 cells was assessed using the Griess protocol. The effects of the combination of ES and SA ethanol extract on RAW 264.7 cell viability were determined using WST-1 (4-[3-(4-Iodophenyl)-2-(4-nitro-phenyl)-2H-5-tetrazolio]-1,3-benzene sulfonate) assay. The levels of proinflammatory cytokines, including interferon-gamma (IFN-γ), tumor necrosis factor-alpha (TNF-α), and interleukin-1 beta (IL-β), as well as the production of inducible nitric oxide synthase (iNOS), were assessed using flow cytometry.

**Results:**

This study demonstrated that ES and SA have excellent NO, iNOS, and proinflammatory inhibitory activities on LPS-induced RAW 264.7 macrophages. The formula ratio of 2ES:1SA showed the best NO inhibitory activity without any cytotoxicity, whereas the higher dose of SA (1ES:2SA) showed the best suppression of iNOS and proinflammatory cytokines IL-1β, IFN-γ, and TNF-α.

**Conclusion:**

The combination of ES and SA ethanol extract could be an alternative agent for reducing excessive inflammation in inflammatory diseases.

## Introduction

Inflammation is related to many diseases, including diabetes and obesity [1], pulmonary fibrosis [2], pre-eclampsia [3], atherosclerosis, and cancer [2]. Inflammation is a complex defense mechanism that maintains homeostasis in the body and combats various infections, injuries, and toxic substances [4]. Inflammatory responses are commonly associated with signaling pathways that modulate several inflammatory mediators in tissue cells and recruit inflammatory cells from the blood to the site of inflammation [5]. Microbial products, such as lipopolysaccharide (LPS), can activate several inflammatory cells, including macrophages and adipocytes, to trigger the production of inflammatory cytokines, such as interleukin 1 beta (IL-1β), tumor necrosis factor-alpha (TNF-α), interferon-gamma (IFN-γ), IL-6, and proinflammatory mediators, such as nitric oxide (NO), prostaglandin E2, and reactive oxygen species. All these components potentially contribute to the prognosis and progression of many inflammatory diseases [6]. Therefore, several potential therapeutic strategies for inflammatory diseases have been developed to inhibit these inflammatory mediators’ production and decrease the inflammatory disease’s prognosis.

Natural products are widely known to be effective in treating several diseases. At present, extensive research has been conducted to develop new drug candidates from natural products to treat inflammation [7]. Several studies have shown that most natural products work more effectively in combination than individually [8–10]. A combination of several agents is expected to have more than one effect. Therefore, in this study, we used a combination of two herbal medicines to reduce inflammation. *Elephantopus scaber* Linn., commonly known as Tapak Liman, is an Asian *Asteraceae* herb widely used as a traditional medicine for several diseases. Several antiviral, anti-inflammatory, antidiuretic, antibacterial, antioxidant, anti-diabetic, and hepatoprotective pharmacological effects of ES have been studied [11, 12]. ES contains several bioactive compounds with anti-inflammatory properties, including deoxyelephantopin and scabertopin [13]. *Sauropus androgynus* (SA) L. Merr, known as Katuk in Indonesia, is a shrub belonging to the *Phyllanthaceae* family that is traditionally used as a breast milk inducer. This plant has several biological properties, including anti-inflammatory and antibacterial properties. Many bioactive compounds such as flavonoids, alkaloids, terpenes, and steroids are found in SA [12]. The previous studies have evaluated the anti-inflammatory effects of combined plant extracts. Asfi *et al*. [10] reported that combination treatment with ES and SA decreased the levels of inflammatory cytokines during bacterial infection in pregnant mice. However, the mechanism underlying the anti-inflammatory properties of these combined plants is unknown.

*In vitro* studies on the inhibitory effect of the combination of ES and SA in LPS-stimulated cells are lacking. It remains unclear whether the anti-inflammatory effect of the combination of ES and SA is associated with inflammatory mediators. Therefore, in the present study, we investigated the suppression effect of the combination of ES and SA ethanol extract on NO production and the production of proinflammatory cytokines (IL-1β, TNF-α, and IFN-γ) in LPS-stimulated RAW 264.7 murine macrophages.

## Materials and Methods

### Ethical approval

This study was based on an *in vitro* experiment; thus, ethical approval was not necessary.

### Study period and location

The study was conducted from May to November 2023 at Laboratory of Animal Physiology, Structure, and Development, Department of Biology, Faculty of Mathematics and Natural Sciences, Brawijaya University, Malang, Indonesia.

### Preparation of ES and SA ethanol extracts

Fresh samples of each plant were purchased from UPT. Laboratorium Herbal Materia Medica Batu, Indonesia, and authenticated by Achmad Mabrur, SKM, M. Kes, head of UPT. Laboratorium Herbal Materia Medica Batu (voucher specimen number: ES: 067/656/102.20/2023 and *S. androgynous*: 067/657/102.20/2023). The leaves of each plant were washed, dried in an oven at 40°C, and ground to a fine powder. The fine powder was macerated in 95% ethanol (1:10) at room temperature (25°C) for 3 days, as described by Christina *et al*. [9] and Christina *et al*. [14]. The mixture was then filtered and evaporated in a rotary evaporator. All extracts were kept air-tight at 4°C until further use.

For cell treatment, 40 mg of ES and SA extract was dissolved in 1 mL dimethyl sulfoxide (EMSURE^®^, Merck KGaA, Darmstadt, Germany) as the extract stock solution. The Dimethyl Sulfoxide concentration was 2% (v/v) when applied to cells. The stock solution was diluted with Dulbecco’s modified Eagle’s medium (DMEM) according to the half maximal inhibitory concentration (IC_50_) and 2× IC_50_ concentration of each extract. The IC_50_ values of ES and *S. androgynous* leaves on RAW 264.7 cells were 3.36 and 88 μg/mL, respectively, which were obtained from our previous study (submitted).

### Cell culture

RAW 264.7 mouse macrophage cells were obtained from Elabscience^®^ (Catalog No.: CL-0190, Elabscience Biotechnology Inc., USA) and maintained in DMEM high glucose (Sigma-Aldrich, Co., Merck KGaA, Darmstadt, Germany) supplemented with 10% fetal bovine serum (certified US, Gibco™, Thermo Fisher Scientific, USA) and 1% 10,000 U/mL penicillin–streptomycin (Gibco™, Thermo Fisher Scientific) at 37°C in a humidified incubator containing 5% carbon dioxide (CO_2_). We used RAW 264.7 cells between passages 20 and 25.

### NO quantification

NO production was evaluated using the Griess reaction method. Briefly, RAW 264.7 cells (1 × 10^5^ cells/mL) were seeded into 24-well plates in complete DMEM for 24 h in a 5% CO_2_ incubator. After 70%–80% confluency, cells were treated with three combinations of ES and SA extracts: 1:1 (IC_50_ ES, 3.36 μg/mL: IC_50_ SA, 88 μg/mL), 1:2 (IC_50_ ES, 3.36 μg/mL: 2× IC_50_ SA, 176 μg/mL), and 2:1 (2× IC_50_ ES, 6.72 μg/mL: IC_50_ SA, 88 μg/mL) for 24 h. To induce inflammation, 5 μg/mL LPS from *Escherichi*a *coli* (Sigma-Aldrich, Co., Merck KGaA) were added to all treated cells for 24 h at 37°C in a humidified incubator containing 5% CO_2_. Untreated control cells without LPS induction were used.

Culture medium was collected after 24 h of treatment for NO analysis using Griess reagent modified (Sigma-Aldrich, Co., Merck KGaA). We used the Griess method based on Dwijayanti *et al*. [15] with slight modifications. A 75 μL culture medium and 75 μL Griess reagent (1:1) were mixed in a 96-well plate and incubated at 25°C for 15 min. The presence of nitrite is indicated by a pink color [16]. The absorbance of each sample was measured at 571 nm using a microplate reader. The nitrite concentration in each sample was determined using a sodium nitrite standard curve. The experiment was performed in six replicates with six different lots of cells.

### Cell viability assay

Cell viability assay was performed using WST-1 (4-[3-(4-Iodophenyl)-2-(4-nitro-phenyl)-2H-5-tetrazolio]-1,3-benzene sulfonate) assay as described by Christina *et al*. [14]. The culture medium was removed and replaced with 150 μL of medium containing 7.5 μL of WST-1 reagent (Roche Diagnostics GmbH, Germany) in each 24-well plate after treatment, as described in the previous NO analysis. Subsequently, the plate was incubated for 30 min in an incubator containing 5% CO_2_. Then, 100 μL solution was aspirated and placed in 96-well plates. The absorbance was measured at 450 nm using a microplate reader. Cell viability was determined using the following formula:


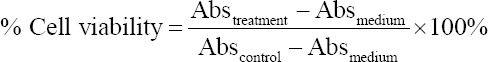


### Measurement of proinflammatory cytokine production

Inhibition of proinflammatory cytokines in LPS-induced RAW 264.7 cells by the ES and SA extract combination was assessed using flow cytometry. RAW 264.7 cells were seeded in 24-well plates at a density of 1 × 10^5^ cells/mL for 24 h in a 5% CO_2_ incubator. Cells were treated with the following treatments for 24 h: (1) negative control, without LPS; (2) positive control, 5 μg/mL LPS; and (4-6) 3 treated group, 5 μg/mL LPS + combination of ES and SA extract: 1:1 (IC_50_ ES, 3.36 μg/mL: IC_50_ ES, 88 μg/mL), 1:2 (IC_50_ ES, 3.36 μg/mL: 2× IC_50_ ES, 176 μg/mL), and 2:1 (2I× IC_50_ ES, 6.72 μg/mL: IC_50_ ES, 88 μg/mL) for 24 h. After incubation for 24 h, treated cells were harvested using 75 μL/well trypsin ethylenediaminetetraacetic acid 0.25% (Gibco™, Thermo Fisher Scientific).

The harvested cells were added to 500 μL phosphate-buffered saline and centrifuged at 769× *g* for 5 min. Next, 50 μL of cell suspension was added to 50 μL of intracellular fixation buffer (eBioscience™, Invitrogen, Thermo Fisher Scientific, USA) and incubated for 20 min at 4°C. We added 500 μL of diluted permeabilization buffer (Invitrogen, Thermo Fisher Scientific) to each sample and centrifuged at 769× *g* for 5 min. Pellets were collected and stained with Fluorescein Isothiocyanate (FITC) IFN-γ antibody, Phycoerythrin (PE) TNF-α antibody, FITC IL-1β antibody, and PE inducible nitric oxide synthase (iNOS) antibody with a dilution ratio of 1:100, respectively. Antibodies were purchased from Santa Cruz Biotechnology, Inc., USA. We measured the relative numbers of IFN-γ, TNF-α, IL-1β, and iNOS using a flow cytometer (BD FACS Calibur™, San Jose, CA, USA) and analyzed them using FlowJo™ version 10 software (Vancouver, BC, Canada). The experiment was performed in six replicates with six different lots of cells.

### Statistical analysis

Data are expressed as mean ± standard deviation and are analyzed using Student’s t-test. All statistical analyses were performed using Microsoft Excel. p < 0.05 and p < 0.01 were considered statistically significant.

## Results

### The combination of ES and SA suppresses NO production in the RAW 264.7 cell line

**Figure-1:**
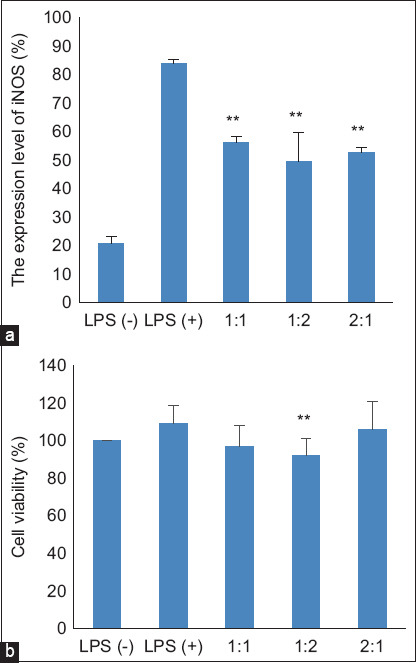
The effects of combination of *Elephantopus* *scaber* and *Sauropus androgynus* on (a) NO production and (b) viability of LPS-induced RAW 264.7 macrophages. The RAW 264.7 cells were treated with LPS and combined extracts for 24 h. The NO levels in the medium were measured by the Griess method, while the viability of cells was measured by WST-1 reagent. The data were observed in triplicate and shown as a means ± standard deviation (SD). * p < 0.05 and ** p < 0.01 versus LPS alone. NO=Nitric oxide, LPS=Lipopolysaccharide, SD=Standard deviation.

NO assay was performed to examine the effect of the combination of ES and SA on NO production in LPS-induced RAW 264.7 macrophages. As shown in Figure-1a, the addition of LPS significantly increased the production of NO (0 ± 0.31 μM vs. 11.21 ± 0.27 μM) in the RAW 264.7 macrophage cell culture. Because NO is a sensitive proinflammatory mediator, an increase in NO concentration in culture media indicates inflammation. The combination of ES and SA significantly suppressed NO production by 4.22 ± 0.53 μM (1:1), 2.65 ± 0.27 μM (1:2), and 0.24 ± 0.23 μM (2:1), respectively. The combination of ES and SA with formula 2ES:1SA resulted in the best inhibition of NO compared with the other formulas.

### Effects of the combination of ES and SA on the cytotoxicity of the RAW 264.7 cell line

To monitor the cytotoxicity of the combination of ES and SA, cell viability was measured using the WST-1 assay. In this study, the combination of 1ES:2SA showed a cell viability of 85%. This value significantly differed from that of the LPS group (Figure-1b). As another combination with a lower dose of SA (1ES:1SA and 2ES:1SA) did not show any cytotoxicity, these results suggest that SA may be cytotoxic when administered at high doses.

### Effect of the ES and SA combination on iNOS protein expression

**Figure-2:**
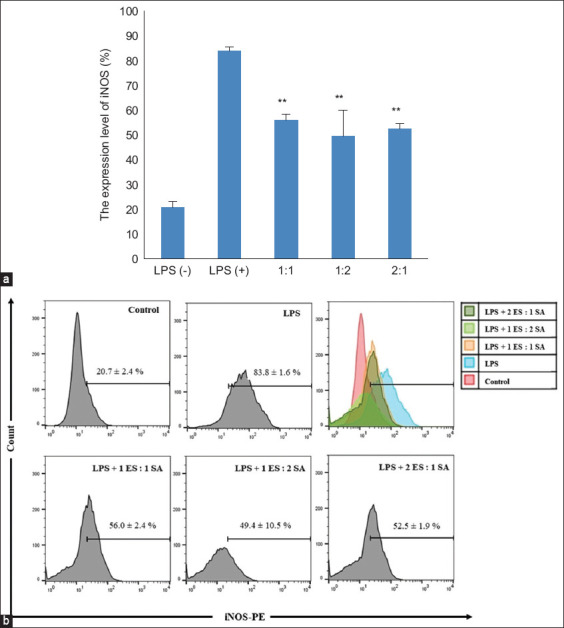
The effects of the combination of *Elephantopus* *scaber* and *Sauropus androgynus* on iNOS protein in LPS-induced RAW 264.7 macrophages. The RAW 264.7 cells were treated with LPS and combined extracts for 24 h. The level of iNOS protein was measured by flow cytometry. (a) The graph showed the mean percentage of iNOS expression ± SD in all treatment groups. (b) The representative histogram from FlowJo™ software (Vancouver, BC, Canada) demonstrated the level of iNOS expression in all group treatments. The data were observed in triplicate. *p < 0.05 and **p < 0.01 versus LPS alone. iNOS=Inducible nitric oxide synthase, LPS=Lipopolysaccharide, SD=Standard deviation.

LPS stimulation of the RAW 264.7 macrophage cell line significantly (p < 0.05) increased iNOS protein expression from 20.7 ± 2.39 % (control) to 83.76 ± 1.6%. This result agrees with the results of the NO measurements. When iNOS, an enzyme that produces NO, increased after exposure to LPS, the product also increased. When the combination of ES and SA extracts was added to LPS-induced RAW 264.7 cell medium, iNOS expression significantly decreased to 56 ± 2.38%, 49 ± 10.46%, and 52.53 ± 1.92%, respectively (Figure-2). From these results, it can be concluded that the optimal combination ratio of 1ES:2SA potentially reduces iNOS expression more than other combination ratios.

### Effects of the combination of ES and SA on the proinflammatory cytokines IL-1β, IFN-γ, and TNF-α

**Figure-3:**
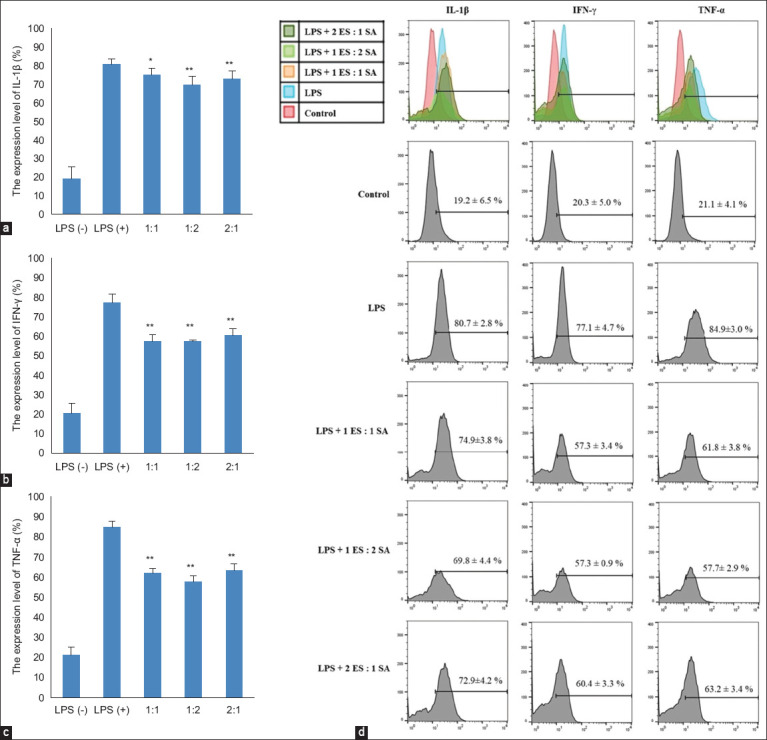
The effects of the combination of *Elephantopus* *scaber* and *Sauropus androgynus* on proinflammatory cytokines (a) IL-1β, (b) IFN-γ, and (c) TNF-α LPS-induced 264.7 macrophages. The RAW 264.7 cells were treated with LPS and combined extracts for 24 h. (d) The level of proinflammatory cytokines was measured by flow cytometry. The data were observed in triplicate and shown as a means ± SD. *p < 0.05 and **p < 0.01 versus LPS alone. IL-1 β=Interleukin-1 beta, IFN-γ: Interferon-gamma, TNF-α=Tumor necrosis factor-alpha, LPS=Lipopolysaccharide, SD=Standard deviation.

LPS-induced RAW 264.7 cells produced more IL-1β cytokines (p < 0.05) than RAW 264.7 cells without LPS treatment (19.15 ± 6.45% vs. 80.68 ± 2.78%). LPS induction for 24 h increased the production of IL-1β cytokines in RAW 264.7 cells. All combinations of ethanol extracts from ES and SA showed reduced IL-1β cytokine levels. However, the addition of 1ES:2SA resulted in a greater reduction in the relative number of IL-1β cytokines compared with the other ratio combinations of ES and SA (Figure-3a and d). The IL-1β levels were 72.28 ± 5.19% (1:1), 69.76 ± 4.36% (1:2), and 72.85 ± 4.18% (2:1).

The relative number of IFN-γ cytokines after LPS induction for 24 h also increased significantly (p < 0.05) compared with the control (20.31 ± 5.03% vs. 77.1 ± 4.68%). The 1ES:1SA and 1ES:2SA combination showed the same effect in reducing the relative number of IFN-γ cytokines after LPS induction for 24 h, which became 57.3 ± 3.4% and 57.3 ± 0.9%, respectively (Figure-3b and d). Similar to the other cytokine parameters, the level of TNF-α also increased from 21.1 ± 4.1% to 84.9 ± 3.0% due to LPS stimulation. The 1ES:2SA combination consistently reduced the relative amount of this cytokine to 57.7 ± 2.9% (Figure-3c and d).

## Discussion

iNOS is a proinflammatory enzyme responsible for NO production [17]. In addition to affecting NO production, LPS-induced inflammation is associated with the upregulation of the proinflammatory cytokines IL-1β [18], IFN-γ [19], and TNF-α [20], which play critical roles in innate immunity and inflammation. LPS induces NO production through various mechanisms. LPS treatment induces NO overproduction by enhancing the expression of iNOS and activating nuclear factor-kappa B (NF-κB), leading to NO release [21]. On the other hand, LPS also induces the production of proinflammatory cytokines, and LPS-induced proinflammatory cytokine expression involves the activation of various signaling pathways. LPS stimulation increases proinflammatory cytokine levels in different cell types, such as human macrophages. This process is mediated through the activation of NF-κB, activator protein-1, and signal transducer and activator of transcription 3 signaling pathways [22, 23].

The results of this study suggest that the combination of ES and SA suppresses NO production in LPS-induced RAW 264.7 cells. In our previous research, the IC_50_ value of ES for suppressing NO production was 6.72 μg/mL, whereas that of SA was 88 μg/mL (under review). When we combined their IC_50_ value dose at the same level (1ES:1SA), it showed a better effect of suppressing approximately 70% NO production (from 11.21% in LPS addition to 4.22% in LPS alone). Interestingly, formula 2ES:1SA showed the best effect in suppressing NO production compared to another formula (0.24%; Figure-1a). Although all formulas showed almost the same potency with respect to suppressed iNOS and proinflammatory cytokines IL-1β, IFN-γ, and TNF-α, formula 1ES:2SA showed the best suppression activity (Figures-2 and 3). However, ES has been traditionally used in folk medicine and has anti-inflammatory properties. Several studies have demonstrated the anti-inflammatory properties of this plant [10, 11]. To support this finding, the previous study by Chan *et al*. [24] has shown that ES is anti-neuroinflammatory by activating Nrf2/HO-1 signaling and inhibiting the p38 MAPK pathway in LPS-induced microglia BV-2 cells. Qi *et al*. [25] demonstrated that the ethanol extract of ES attenuates the inflammatory response by inhibiting NF-κB signaling by dampening p65–DNA binding activity. Lactone and sesquiterpenoids exhibit significant anti-inflammatory activity against ES [26]. The active compounds in ES that are responsible for its anti-inflammatory properties include scabertopin, isoscabertopin, deoxyelephantopin and isodeoxyelephantopin [26].

Research on the molecular mechanism underlying the anti-inflammatory activity of SA is limited. However, it has been reported that the plant exhibits anti-inflammatory properties, possibly due to its bioactive phytochemicals. Ethanolic extracts of SA exhibit anti-inflammatory activity, which may be attributed to bioactive phytochemicals such as flavonoids, saponins, and alkaloids [27–29]. Although the anti-inflammatory activity of SA has been demonstrated, Bunawan *et al*. [27] reported that excessive consumption of SA could cause drowsiness, constipation, and bronchiolitis obliterans, which may lead to respiratory failure. The cytotoxicity of this agent may be due to papaverine, an alkaloid present in SA. Because 1ES:2SA was more potent than other formulas in inhibiting iNOS and proinflammatory cytokines, this study demonstrated the anti-inflammatory mechanisms of SA. Although the specific molecular mechanisms are not fully elucidated, the anti-inflammatory effects of SA could be linked to the modulation of inflammatory pathways and the regulation of inflammatory mediators.

## Conclusion

All combinations of ES and SA exhibited excellent NO, iNOS, and proinflammatory inhibition activity in LPS-induced RAW 264.7 macrophages. The combination with a higher dose of ES (2ES:1SA) resulted in the best NO inhibitory activity without cytotoxicity, whereas a higher dose of SA (1ES:2SA) resulted in the best suppression of iNOS and the proinflammatory cytokines IL-1β, IFN-γ, and TNF-α. Further research is needed to elucidate the specific molecular mechanism underlying the anti-inflammatory effects of this combination plant and to identify the compounds responsible for this mechanism.

## Authors’ Contributions

MSD: Substantial contributions to the conception and design of the study. YIC: Performed the data acquisition and revised the manuscript. DRD: Performed data interpretation and revised the manuscript. SR: Performed data analysis and contributed to study design. MSD, YIC, DRD and SR: Drafted the manuscript. All authors have read, reviewed, and approved the final manuscript.
